# Human microbiomes and their roles in dysbiosis, common diseases, and novel therapeutic approaches

**DOI:** 10.3389/fmicb.2015.01050

**Published:** 2015-10-06

**Authors:** José E. Belizário, Mauro Napolitano

**Affiliations:** Department of Pharmacology, Institute of Biomedical Sciences, University of São Paulo, São PauloBrazil

**Keywords:** microbiome, metagenomics, phage therapy, CRISPR/Cas system, quorum sensing, pharmacomicrobiomics

## Abstract

The human body is the residence of a large number of commensal (non-pathogenic) and pathogenic microbial species that have co-evolved with the human genome, adaptive immune system, and diet. With recent advances in DNA-based technologies, we initiated the exploration of bacterial gene functions and their role in human health. The main goal of the human microbiome project is to characterize the abundance, diversity and functionality of the genes present in all microorganisms that permanently live in different sites of the human body. The gut microbiota expresses over 3.3 million bacterial genes, while the human genome expresses only 20 thousand genes. Microbe gene-products exert pivotal functions via the regulation of food digestion and immune system development. Studies are confirming that manipulation of non-pathogenic bacterial strains in the host can stimulate the recovery of the immune response to pathogenic bacteria causing diseases. Different approaches, including the use of nutraceutics (prebiotics and probiotics) as well as phages engineered with CRISPR/Cas systems and quorum sensing systems have been developed as new therapies for controlling dysbiosis (alterations in microbial community) and common diseases (e.g., diabetes and obesity). The designing and production of pharmaceuticals based on our own body’s microbiome is an emerging field and is rapidly growing to be fully explored in the near future. This review provides an outlook on recent findings on the human microbiomes, their impact on health and diseases, and on the development of targeted therapies.

## Introduction

The evolution of *Homo sapiens* is linked to a mutualistic partnership with the human gut microbiota. The human genome is part of a collective genome of complex commensal, symbiotic, and pathogenic microbial community that colonizes the human body. Our microbiome includes not only bacteria, but also viruses, protozoans, and fungi (Backhed et al., 2012). Bacteria are a vast group of living organisms considered a domain of life in themselves (Woese et al., 1990). They are classified using DNA-based tests, morphologically and biochemically based on cell wall type, cell shape, oxygen requirements, endospore production, motility, and energy requirements. Hans Christian Gram (1850–1938), a Danish scientist, discovered that the presence of high levels of peptidoglycan (50–90%) produced a dark violet color, while low levels (<10%) resulted in reddish/pinkish colors, which are the respective staining of Gram-positive and Gram-negative bacteria. The Gram-negative cell wall is also characterized by the presence of lipopolysaccharides (LPSs). Based on their capacity to produce energy in presence or absence of oxygen, bacteria can also be classified as aerobic, anaerobic or facultative anaerobic. In addition to the generation of ATP via aerobic or anaerobic respiration, bacteria can also produce energy via fermentation. Facultative anaerobic bacteria are able to generate ATP with or without oxygen, while obligated anaerobic bacteria do not tolerate it and only survive in anaerobiosis. *Lactobacillus, Staphylococcus*, and *Escherichia coli* are examples of facultative anaerobic bacteria. *Bacteroides*, on the other hand, are obligated anaerobic species. In inflamed tissues, the enterocytes produce reactive oxygen species (ROS) and kill anaerobic bacteria increasing the abundance of aerobic and facultative species.

Bacteria are classified phylogenetically based on the analysis of nucleotide sequences of small subunit ribosomal RNA operons, mainly variable regions of the bacterial specific ribosomal RNA, 16S rRNA (Woese, 1987; Woese et al., 1990; Marchesi et al., 1998). Currently, the Bacteria domain is divided into many phyla; however, the majority of microbes forming the human microbiota can be assigned to four major phyla: Firmicutes, Bacteroidetes, Actinobacteria, and Proteobacteria (Zoetendal et al., 2008; Arumugam et al., 2011; Segata et al., 2012). Firmicutes and Bacteroidetes represent more than 90% of the relative abundance of the gut microbiome (Arumugam et al., 2011; Segata et al., 2012). Firmicutes are a diverse phylum composed mainly of the Bacilli and Clostridia classes. They are Gram-positive, anaerobic (Clostridia) and obligate or facultative aerobes (Bacilli) characterized by a low GC content. Bacteria of *Clostridium* species produce endospores in order to survive to adverse (aerobic) conditions (Paredes-Sabja et al., 2014). The phylum Bacteroidetes is composed of Gram-negative, non-spore forming anaerobic bacteria that tolerate the presence of oxygen but cannot use it for growth. Actinobacteria (e.g., *Bifidobacterium)* are Gram-positive, multiple branching rods, non-motile, non-spore-forming, and anaerobic bacteria. Proteobacteria (e.g., *Escherichia*, *Klebsiella, Enterobacter*) are aerobic or facultative anaerobic, Gram-negative, non-spore-forming rod bacteria, which inhabit the intestinal tract of all vertebrates.

Recent survey studies on the variation of human microbiomes concluded that European individuals could be classified in up to three enterotypes based on 16S rRNA gene data and functional metagenome (whole genome shotgun) data (Arumugam et al., 2011; Koren et al., 2013). An enterotype refers to the relative abundance of specific bacterial taxa within the gut microbiomes of humans. The functional metagenome of each enterotype revealed differences in the proportions of genes involved in carbohydrate versus protein metabolism, which is consistent with diets of different populations (Arumugam et al., 2011; Koren et al., 2013). People differ by species composition, distribution, diversity, and numbers of bacteria (Yatsunenko et al., 2012). The dietary habits are the critical contributing factor. Diversity (microbiome variation and complexity) increases from birth and reaches its highest point in early adulthood, thereafter declining with old age. However, larger longitudinal studies that include more populations, such as South Americans, Indians and Africans need to be done to identify the actual structure and biological impact of the distinct human microbiomes. These studies may also reveal how evolution of life-styles modulated ancestral and modern human microbiomes. Here we will present and discuss recent advances of microbiome studies and the strategies for the development of innovative pharmaceuticals based on emerging population and individual microbiota genomic information.

## Metagenomics

The recent development of next generation sequencing (NGS) technologies such as 454, Solexa/Illumina, Ion Torrent and Ion Proton sequencers and the parallel expansion of powerful bioinformatics programs made possible the genomic analysis of over 1,000 prokaryotic and 100 eukaryotic organisms, including over 1,200 complete human genomes (Flintoft, 2012; Belizario, 2013). Metagenomics is a biotechnological approach to study genomic sequences of uncultivated microbes directly from their natural sources (Wooley et al., 2010). This allows the simultaneous analysis of microbial diversity connecting it to specific functions in different environments, such as soil, marine environments, and human body habitats (Ley et al., 2008; Robinson et al., 2010; Culligan et al., 2014). Using these novel methods, scientists have provided evidence for the existence of more than one thousand microorganism species living in our body (Arumugam et al., 2011; Segata et al., 2012) and an estimation of 10^7^ to 10^9^ different species of bacteria living on earth (Curtis et al., 2002). More important, the metagenomics approach has the potential to uncover entirely novel genes, gene families, and their encoded proteins, which might be of biotechnological and pharmaceutical relevance.

Currently several international projects aimed at the characterization of the human microbiota are being carried out. The Human Microbiome Project (HMP) is a research initiative of the National Institute of Health (NIH) in the United States, which aims to characterize the microbial communities found in several different sites of the human body (Turnbaugh et al., 2007; Backhed et al., 2012; Human Microbiome Project, 2012a,b). MetaHIT (Metagenomics of the Human Intestinal Tract) is a project financed by the European Commission and is under management of a consortium of 13 European partners from academia and the industry. The International Human Microbiome Consortium (IHMC) is composed of European, Canadian, Chinese, and US scientific institutions^1^.

A simple molecular approach to explore the microbial diversity is based on the analysis of variable regions of 16S rRNA gene using “universal” primers which are complementary to highly conserved sequences among the homologous 16S rRNA genes (Marchesi et al., 1998; Culligan et al., 2014). These genes contain nine hypervariable regions (V1–V9) whose sequence diversity is appropriated for characterizing bacterial community compositions in complex samples (Guo et al., 2013; Jiang et al., 2014; Montassier et al., 2014). DNA sequences obtained with this approach can be mapped onto a reference set of known bacterial genomes. For this purpose useful bioinformatics tools and databases are available. For example, the SILVA database^2^ is a comprehensive online resource for quality checked and aligned ribosomal RNA sequence data that helps determine an optimal alignment for the different sequence regions (Pruesse et al., 2007). First released in 1995, The Ribosomal Database Project (RDP) is another database that provides high quality alignments of archaeal and bacterial 16S rRNA sequences as well as fungal 28S rRNA sequences (Maidak et al., 1996). The microbial profiling and phylogenetic clustering of microbiomes of the American and European population have been already deposited and are free for consultation^3^^,^^4^. The HMP projects and other independent projects have been generating an enormous amount of metagenomic data and the assemblies of microbiome data is being undertaken by the Genomes OnLine Database^5^. The data management system for cataloging and continuous monitoring of worldwide sequencing projects contains data from over 4000 metagenome sequencing projects, in which more than 1500 are aimed at the characterization of host associated metagenomes (Human Microbiome Jumpstart Reference Strains et al., 2010; Fodor et al., 2012; Reddy et al., 2015).

**FIGURE 1:**
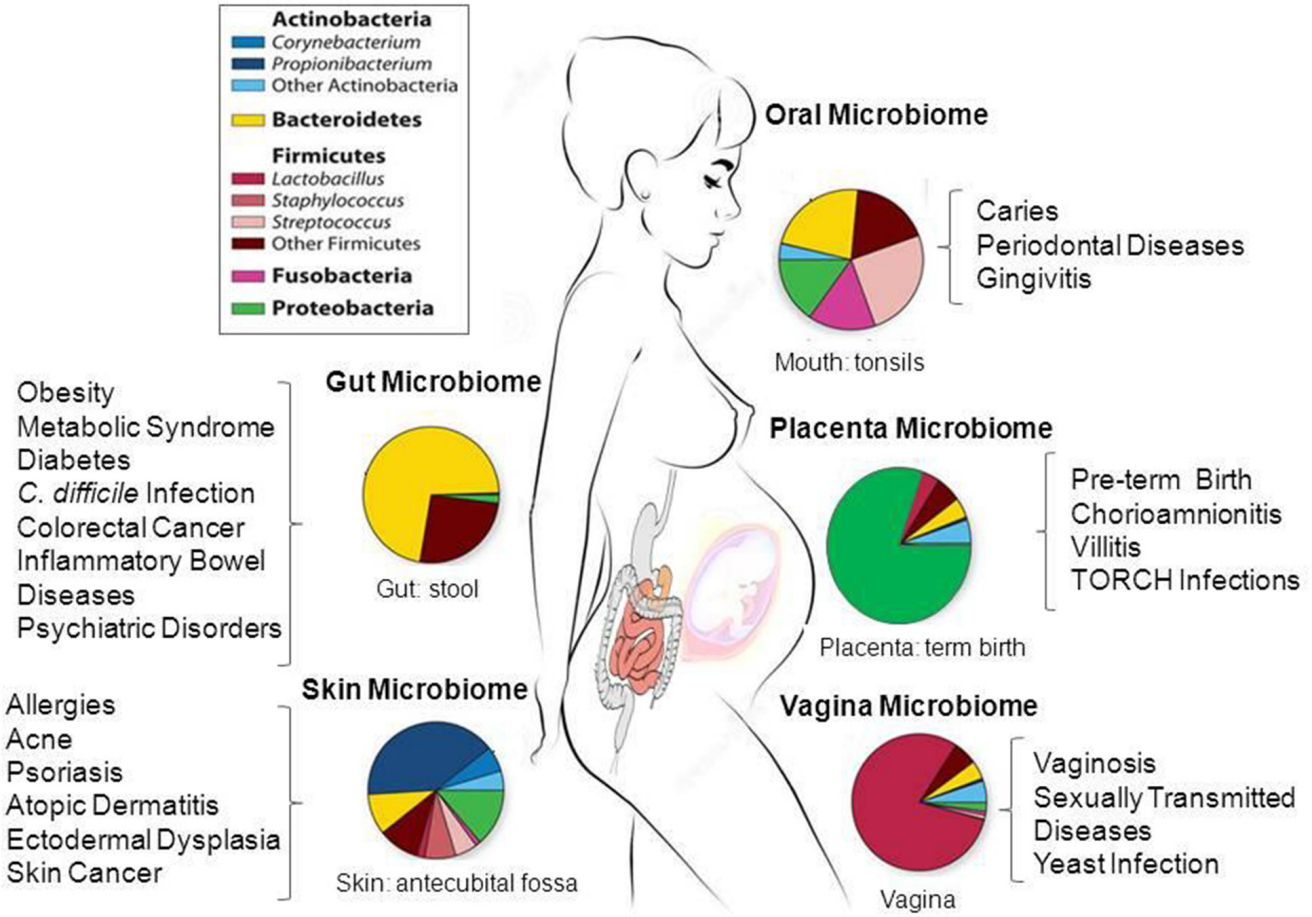
**Taxonomic distribution, prevalence and abundance of microbial taxa that inhabit healthy human body sites as defined in the human microbiome projects (HMP).** The colored rectangles denote phylum/class and genera. Clinical studies of the microbiome will help to elucidate the link between microbes and the promotion of a large number of diseases and pathological conditions as shown in the figure. The images were adapted from NIH HMP (http://www.hmpdacc.org/) and National Human Genome Research Institute (https://www.genome.gov/). TORCH, Toxoplasmosis, Oher infections (coxsackievirus, HIV, syphilis, etc), Rubella, Cytomegalovirus, Herpes simplex.

The first release of the HMP database included microbiome data of nasal passages, the oral cavity, skin, gastrointestinal tract, and urogenital tract (Human Microbiome Project, 2012a,b). **Figure 1** schematically summarizes the data of these studies. The results of over 690 human microbiomes have shown that the majority of bacteria of the gut microbiome belongs to four phyla: Firmicutes, Bacteroidetes, Actinobacteria, and Proteobacteria (Human Microbiome Project, 2012a,b). Only a fraction of microbes identified so far have been successfully cultured, and thousands are yet to be fully sequenced for a deeper taxonomic resolution (strains and subspecies) and functional analysis at the genomic level (Qin et al., 2010; Robinson et al., 2010; Abubucker et al., 2012; Flintoft, 2012; Zhou et al., 2013).

The metagenome wide association studies in development in many countries are promising in predicting new diagnostic and prognostic tools for numerous human disorders. The results of these studies will dramatically increase our knowledge of diseases linked to microbial composition (Qin et al., 2010; Clemente et al., 2012; Flintoft, 2012; Gevers et al., 2012). In order to better understand the host-gene-microbial interactions and the role of non-pathogenic and pathogenic strains in large populations, we need to compare microbiome profiles across multiple body sites and microbiome datasets under environmentally controlled normal and disease conditions. In the following sections, we will provide a synthesis of the recent studies on the human microbiomes identified in some major body sites.

### Gut Microbiome

The HMPs have shown that the human gut harbors one of the most complex and abundant ecosystems colonized by more than 100 trillion microorganisms (Human Microbiome Project, 2012a,b). In adults, the majority of the bacteria found in the gut belong to two bacterial phyla, the gram-negative Bacteroidetes and the gram-positive, Firmicutes; and the others represented at subdominant levels are the Actinobacteria, Fusobacteria, and Verrucomicrobia phyla, but this varies dramatically among individuals (Eckburg et al., 2005; Zoetendal et al., 2008; Arumugam et al., 2011; Backhed et al., 2012; Segata et al., 2012). For instance, the most abundant genera from the Bacteroidetes phylum are *Bacteroides* and *Prevotella species*, which represent 80% of all Bacteroidetes in fecal samples. Nonetheless, many of the taxa numerically underrepresented and less-abundant bacterial species exert fundamental functions at a particular location in the gut. To better define these different microbial colonization and microbiota structure in different cohorts arose the concept of ‘enterotype clusters’ that allow the classification of each individual based on the relative abundance of specific bacterial taxa in fecal samples, and their microbial metabolic and functional pathways (Arumugam et al., 2011; Backhed et al., 2012; Koren et al., 2013). The results of metagenomic sequencing of fecal samples from European, American, and Japanese subjects confirmed the three robust clusters dominated by *Bacteroides* (enterotype 1), *Prevotella* (enterotype 2), and *Ruminococcus* (enterotype 3), each one characterized by specific taxonomic composition and relative abundance of metabolic pathways. For example, enterotype 1 was enriched in biosynthesis of biotin, riboflavin pantothenate and ascorbate; enterotype 2 in biosynthesis of thiamine and folate. Enterotype 3 showed high abundance of genes involved in haem biosynthesis pathway. Although in one of these studies (Arumugam et al., 2011) it was confirmed that a set of 12 genes correlated with age and a set of three functional modules with the body mass index (BMI), further studies will be required to determine if specific microbiome and/or enterotype is associated with gender, BMI, health status, diet, and age of individuals (Arumugam et al., 2011; Backhed et al., 2012; Koren et al., 2013).

Although stable over long periods, the composition and functions of the gut microbiome may be influenced by a number of factors including genetics, mode of delivery, age, diet, geographic location, and medical treatments (Clemente et al., 2012; Brown et al., 2013). The intestinal microbiota is acquired in the postnatal periods of time, consisting of a wide variety of bacteria that plays different functions in the human host, including nutrient absorption, protection against pathogens, and modulation of the immune system (Brown et al., 2013). The gut is an anaerobic environment in which indigenous species have co-evolved with the host. The aerobic pathogenic species cannot invade and colonize it; however, anaerobic and facultative pathogenic species can invade it causing diseases. High diversity defines healthy human gut microbiomes, whereas reduction in diversity may be associated with dysbiosis (Manichanh et al., 2006; Backhed et al., 2012). Dysbiosis refers to an imbalance in the microbiome structure that results from an abnormal ratio of commensal and pathogenic bacterial species. Many studies have suggested a possible direct relationship between dysbiosis and inflammatory and metabolic diseases such as is inflammatory bowel diseases (IBD) including colitis and Crohn’s disease (CD), obesity and cancer (Clemente et al., 2012; Sartor and Mazmanian, 2012; Brown et al., 2013). However, investigation of such a complex ecosystem is difficult and it is still not easy to define how shifts in microbial composition and member abundance can lead to diseases. Induction of some IBD has been linked to a reduction of Firmicutes and Bacteroidetes and an expansion of Proteobacteria. For example, *Faecalibacterium prausnitzii*, a prominent member of Clostridium group IV (Firmicutes), protective and anti-inflammatory commensal bacterium, is frequently reduced in CD patients (Sokol et al., 2008; Sartor and Mazmanian, 2012). Despite these advances, it should be noted that microbiota composition varies between different locations in the gastrointestinal tract (Eckburg et al., 2005; Zoetendal et al., 2008; Arumugam et al., 2011; Cucchiara et al., 2012; Segata et al., 2012; Lepage et al., 2013). Most studies in the literature have explored only fecal microbiota. Fecal samples contain between 1,000 and 1,150 bacterial species, and up to 55% are uncultivable and thus uncharacterized (Zoetendal et al., 2008; Qin et al., 2010; Segata et al., 2012; Zhou et al., 2014). Our knowledge is especially limited when it comes to the other parts of the GI tract, a potential source of uncharacterized microbial species, which is largely due to sampling constraints.

The dysregulation of the intestinal immune system can also trigger microbial dysbiosis (Clemente et al., 2012; Sartor and Mazmanian, 2012; Brown et al., 2013). Many different inflammatory diseases are characterized by mutations or loss of some innate response genes in lymphoid tissues, Paneth cells, smaller Peyer’s patches and mesenteric lymph nodes (Clemente et al., 2012; Frantz et al., 2012; Sartor and Mazmanian, 2012). The growth of microbiota communities is under control of distinct subfamilies of host genes encoding antimicrobial peptides (AMPs). AMPs are the most ancient component of the innate host response against bacterial infections (Guani-Guerra et al., 2010; Ostaff et al., 2013). When bacteria colonize a given human habitat, the expression of AMPs, including α and β defensins and cathelicidins, is upregulated in order to limit the spreading of bacteria. The equilibrium between the immune system and immunoregulatory functions of bacteria appears to be a delicate balance in which the loss of a specific species can lead to an overreaction or suppression of the innate immune system (Round and Mazmanian, 2009; Clemente et al., 2012; Sartor and Mazmanian, 2012; Brown et al., 2013). Intestinal epithelial cells (IECs) form a physical and immunological barrier that separate luminal bacteria from underlying immune cells in the intestinal mucosa. IECs and hematopoietic cells express a variety of receptors called pattern recognition receptors (PRRs) that mediate the interactions between the immune system and the commensal microbiota (Frantz et al., 2012). Toll-like receptors (TLRs) and nuclear oligomerization domain-like receptors (NLRs) are examples of PRR that recognize unique microbial molecules named microbe-associated molecular patterns (MAMPs) including lipopolysaccharides (LPS), lipid A, peptidoglycans, flagella, and microbial RNA/DNA. These receptors activate inflammasomes and thereby the production of cytokines TNF-α and IL-1β (Brown et al., 2013; Sangiuliano et al., 2014). Myeloid differentiation factor MyD88 is an adaptor protein that is essential for TLRs signaling and host-microbial interactions and tissue homeostasis (Sangiuliano et al., 2014). Mice lacking MyD88 in IECS (IEC-Myd88-/- mice) display intestinal barrier disruption, deficiency in the production of pro-inflammatory cytokines and AMPs and overgrowth of several enteric bacterial pathogens (Frantz et al., 2012). It will be important to understand when and how dysbiosis and genetic defects in mucosa-IECs and innate regulatory mechanisms can lead to development of infectious or inflammatory diseases.

Microorganisms synthesize a wide range of low-molecular weight signaling molecules (metabolites), many of which are similar to metabolites produced by human cells (Wikoff et al., 2009). The maintenance of a stable, fermentative gut microbiota requires diets rich in whole plant foods particularly high in dietary fibers and polyphenols (Zoetendal et al., 2008). Under anaerobic conditions, species belonging to the *Bacteroides* genus, and to the Clostridiaceae and Lactobacillaceae families, produce short-chain fatty acids (SCFAs). Acetate (with two carbons), propionate (with three carbons), and butyrate (with four carbons) are SCFA used by the epithelial cells of the colon (colonocytes) and act as a major player in maintenance of gut homeostasis (Meijer et al., 2010). SCFAs induce the secretion of glucagon-like peptide (GLP-1) and peptide YY (PYY), which increase nutrient absorption from the intestinal lumen. This is a key process in controlling mucosal proliferation, differentiation and maintenance of mucosal integrity (Round and Mazmanian, 2009). Individuals colonized by bacteria of the genera *Faecalibacterium, Bifidobacterium, Lactobacillus, Coprococcus, and Methanobrevibacter* have significantly less of a tendency to develop obesity-related diseases like type-2-diabetes and ischemic cardiovascular disorders (Ley et al., 2006; Le Chatelier et al., 2013). These species are characterized by high production of lactate, propionate and butyrate as well as higher hydrogen production rates, which are known to inhibit biofilm formation and activity of pathogens, including *Staphylococcus aureus*, in the gut (Le Chatelier et al., 2013). Genetic and diet-induced mouse models of obesity have shown that the Bacteroidetes/Firmicutes ratio is decreased in obese animals compared to non-obese animals, which is consistent with what has been observed in human obese subjects (Ley et al., 2005, 2006; Le Chatelier et al., 2013; Verdam et al., 2013). However, controversies exist regarding the human data on gut microbiota composition in relation to obesity (Turnbaugh et al., 2009; De Filippo et al., 2010; Verdam et al., 2013). The intestinal microbiota changes in obese mice may increase the intestinal permeability and inflammation locally and in adipose tissues (Cani and Delzenne, 2011; Kootte et al., 2012). As discussed in recent studies, the microbial-derived LPS released through circulation may promote low-grade inflammatory process and metabolic disturbances related to obesity, such as insulin resistance and type-2 diabetes (Cani and Delzenne, 2011; Kootte et al., 2012). Despite our currently incomplete understanding of the mechanisms, there have been high expectations that targeted changes in microbiota by the rational use of prebiotics and probiotics might abolish metabolic alterations associated with obesity (Cani and Delzenne, 2011; Kootte et al., 2012).

### Vagina Microbiome

The first study based on pyrosequencing of barcoded 16S rRNA genes of vaginal microbiota performed on samples from North-American women revealed the inherent differences within and between women in different ethnic groups (Ravel et al., 2011). The vaginal microbial composition from three vaginal sites (mid-vagina, cervix, and introitus) has been compared to the buccal mucosa and the perianal region in recent studies (Fettweis et al., 2012; Romero et al., 2014; Vaginal Microbiome consortium^6^). These studies have shown that the vagina possesses over 200 phylotypes and that the most predominant belong to the phyla Firmicutes, Bacteroidetes, Actinobacteria, and Fusobacteria (Ravel et al., 2011; Romero et al., 2014). The vagina has low pH due to secretion of lactic acid and hydrogen peroxide by *Lactobacillus* sp. If *Lactobacillus* decreases under the effects of antibiotics, *Gardnerella vaginalis* and *Peptostreptococcus anaerobius*, *Prevotella* sp., *Mobiluncus* sp. *Sneathia*, *Atopobium vaginae*, *Ureaplasma*, *Mycoplasma*, and numerous fastidious or uncultivated anaerobes can cause bacterial vaginosis (BV). BV is an ecological disorder of the vaginal microbiota that affects millions of women annually, and is associated with numerous adverse health outcomes including preterm birth and acquisition of sexually transmitted infections, e.g., HIV, *Neisseria gonorrhoeae*, *Chlamydia trachomatis*, and HSV-2 (Kenyon et al., 2013). *Lactobacillus* morphotypes predominate in normal grade 1. BVs grade 3 and higher are characterized by a reduced number of lactobacilli and increased diversity, especially high concentration of Gram-negative bacteria and coccobacillus (e.g., *G. vaginali*s and *G. mobiluncus)* and *Peptostreptococcus* (Delaney and Onderdonk, 2001). The results of microbiome studies of the vagina are showing different patterns and imbalances in bacterial communities associated with BVs, as well as those associated with non-infectious pathological states that predict increased risk for infertility, spontaneous abortion, and preterm birth.

### Oral Microbiome

Advances in microbiological diagnostic techniques have shown the complex interaction between the oral microbiota and the host (Segata et al., 2012; Jiang et al., 2014; Perez-Chaparro et al., 2014). Bacteria, fungi, archaea, viruses, and protozoa are part of the oral microbiome. The HMP investigated bacterial communities in nine intraoral sites: buccal mucosa, hard palate, keratinized gingiva, palatine tonsils, saliva, sub-and supra gingival plaque, throat, and tongue dorsum (Human Microbiome Project, 2012a,b). Over 300 genera, belonging to more than 20 bacterial phyla were identified (Zhou et al., 2013; Jiang et al., 2014). However, only a limited number of species find proper conditions to colonize the root canal system (Zhou et al., 2013; Perez-Chaparro et al., 2014). The microbiota of periodontitis or caries is usually complex consisting of Gram-negative anaerobic bacteria such as *Porphyromonas gingivalis, Treponema denticola, Prevotella intermedia, Tannerella forsythia*, and *Agregatibacter actinomycetemcomitans* (Mason et al., 2013; Jiang et al., 2014). Most early data on the endodontic microbiota were obtained by culture-based method and it is likely that not-yet-cultivable and unknown species of bacteria play a role in oral microbial shift toward a disease (Mason et al., 2013; Zaura et al., 2014). As expected, deep DNA sequencing data revealed a larger number of taxa involved in endodontic infections. Species of phyla Bacteroidetes, Firmicutes, Proteobacteria, Spirochaetes, Synergistetes, and *Candidatus Saccharibacteria* were more frequently found. All these studies on bacterial diversity in endodontic infections revealed high inter-subject variability, indicating the need for further studies using homogenous diagnosis criteria in a significant number of healthy subjects (Mason et al., 2013; Jiang et al., 2014; Perez-Chaparro et al., 2014).

### Skin Microbiome

The skin is the human body’s largest organ, colonized by over 100 microbial phylotypes, most of which are harmless or even beneficial to their host (Rosenthal et al., 2011; Ladizinski et al., 2014; Zhou et al., 2014). Phylotypes, microbial abundance and diversity differ in relation to skin color, race, and geographic location (Grice et al., 2009; Rosenthal et al., 2011). Colonization is influenced by the ecology and the epidermis layers of the skin surface. Therefore it is highly variable depending on topographical location, endogenous host factors and exogenous environmental factors. The Actinobacteria phylum is the most abundant on the skin. Gram-positive *Staphylococcus epidermidis* and *Propionibacterium acnes* are predominant on human epithelia and in sebaceous follicles, respectively. *Propionibacterium acnes* colonizes healthy pores and is responsible for the production of SCFAs and thiopeptides, which inhibit the growth of *Staphylococcus aureus* and *Streptococcus pyogenes*. However, depending on the host’s immune system, the overgrowth and clogging of pores allow subsequent colonization of *S. epidermidis* and *Staphylococcus aureus.* Atopic dermatitis is one chronic inflammatory condition of the skin that occurs in many children and adults (Grice et al., 2009). *Staphylococcus* sp. *Corynebacterium* sp. and the fungi *Candida* sp, and *Malassezia* sp. are also frequently associated with a number of skin diseases, including atopic dermatitis and abnormal flaking and itching of the scalp (Grice et al., 2009).

The skin microbiota is under autonomous control of the local cutaneous immune system, thus it is independent of the systemic immune response which is modulated by the gut microbiota (Naik et al., 2012). The major innate mechanism of antimicrobial defense on the skin consists of AMPs, for example defensins, cathelicidin LL-37 and dermcidin (Guani-Guerra et al., 2010). These peptides are emerging as important tools in the control of skin pathogenic bacteria as well as bacteria involved in diseases of the lung and gastrointestinal tract. Many AMPs bind to the phospholipid membrane surfaces, forming ion-channels and pores causing leakage and cell death (Guani-Guerra et al., 2010; Ostaff et al., 2013). However, their specific immunomodulatory roles in innate immune defense against bacterial and viral infection remain poorly understood (Ostaff et al., 2013; Wang, 2014). An enhanced understanding of the skin microbiome is necessary to gain insight into AMPs and innate response in human skin disorders. The cutaneous inflammatory disorders such as atopic dermatitis, psoriasis, eczema, and primary immunodeficiency syndromes have been associated with dysbiosis in the cutaneous microbiota. The skin commensals promote effector T cell response, via their capacity to control the NF-κB signaling and the production of cytokines TNF-α and IL-1β (Hooper et al., 2012). The binding of the skin microbiota components to TLRs or NLRs allows a sustainable homeostasis toward innate and adaptive immunity within a complex epithelial barrier throughout distinct topographical skin sites.

### Placenta Microbiome

Historically, the fetus and intrauterine environment were considered sterile. However, the first profile of microbes in healthy term pregnancies identified a unique microbiome niche in normal placenta, composed of non-pathogenic commensal microbiota from the Firmicutes, Tenericutes, Proteobacteria, Bacteroidetes, and Fusobacteria phyla (Aagaard et al., 2014). This study describes the microbial communities of 320 placental specimens and, despite the expected differences between individuals, the taxonomic classification of the placental microbiome bears most similarity to the non-pregnant oral microbiome, in particular to those associated with tongue, tonsils, and gingival plaques. One predominant species was *Fusobacterium nucleatum*, a Gram-negative oral anaerobe. *E. coli* was also found in placenta; however, it is not present in the oral microbiome (Aagaard et al., 2014). The authors suggested a possible hematological spread of oral microbiome during early vascularization and placentation. The pathways related with the metabolism of cofactors and vitamins were the most abundant among placental functional gene profiles, which is different from the metabolic pathways found in other body sites (Aagaard et al., 2014).

The balance of the different microbe species in and on the human body changes throughout life and particularly in different stages of pregnancy (Qin et al., 2010; Human Microbiome Project, 2012a,b). It is well known that preterm delivery (<37 weeks) causes substantial neonatal mortality and morbidity (DiGiulio et al., 2008). Placentas from normal deliveries and preterm deliveries contained different populations of microbial species (Groer et al., 2014). The gram-negative bacillus *Durkholderia* was associated with preterm delivery and the gram-positive, rod-shaped, facultative anaerobic bacteria *Paenibacillus* with term delivery (Aagaard et al., 2014). Consistent with other studies, an enrichment in Streptococci, *Acinetobacter* and *Klebsiella* was also demonstrated in women with history of antenatal infection (Aagaard et al., 2014).

The presence of different microbes in amniotic fluid, umbilical cord blood, meconium (first stool), placental and fetal membranes suggested the existence of various routes and mechanisms by which bacteria from different microbiota translocate to placenta and babies (DiGiulio et al., 2008). Studies in mice have demonstrated the placental transmission from mother’s oral microbiota (Fardini et al., 2010). Many of these organisms are transmitted to babies during nursing. Babies born vaginally have more diverse gut microbial communities similar to their mother’s vaginal microbiota, while microbiomes of babies delivered by Cesarean section are similar to skin microbiota (Dominguez-Bello et al., 2010). The lack of exposure to maternal vaginal microbiome might explain why cesarean section babies are at greater risk of developing type 1 diabetes, celiac disease, asthma, and obesity (DiGiulio et al., 2008; Dominguez-Bello et al., 2010). Breastfed babies’ microbiome is enriched with *Lactobacillus* and *Bifidobacterium* species whereas microbiome of babies fed with formula/solid food are enriched with Enterococci, Enterobacteria, Bacteroides, Clostridia, and Streptococci (Guaraldi and Salvatori, 2012; Palmer et al., 2012; Thompson et al., 2015). The transition from breast milk to solid foods is associated with acquisition of a more adulthood-like microbiome; however, infectious diseases, antibiotic use and the characteristics of the diet can interfere with babies’ microbiota composition (Thompson et al., 2015). Together, these findings emphasize the need for further studies on placental microbiome for elucidating more mechanisms to be explored in the prevention and treatment of babies from preterm birth and other diseases.

## Microbiota-based Pharmaceuticals

Metagenomics has proven to be a powerful tool in determining the diversity and abundance of microbes in the human body. The microbiome databases have been explored as sources of interesting targets to drug development (Cani and Delzenne, 2011; Collison et al., 2012; Haiser and Turnbaugh, 2012; Carr et al., 2013; Wallace and Redinbo, 2013). Therapeutic interventions in the microbiome can be directed against molecular entities, such as essential and antibiotic resistance genes to quorum sensing systems components used to control microbial networking behaviors, including the chemical communication and production of virulence factors (Collison et al., 2012). In the next sections, we will present and discuss strategies to discover novel antimicrobial targets as well as dietary interventions and microbial modification genetic tools to eliminate pathogenic microorganisms and to control dysbiosis.

### Targeting Essential Genes

Searching of essential genes for bacterial growth and viability is the first step for identifying potential drug targets (Wallace and Redinbo, 2013). Computational analyses can provide candidate targets in microbial community of pharmacological significance for controlling bacterial species involved in chronic diseases, metabolic, and cardiovascular diseases as well as drug metabolism (Collison et al., 2012). The metagenomic databases are critical for constructing gene and protein networks and an initial framework for drug target screening (Collison et al., 2012; Carr et al., 2013; Manor and Borenstein, 2015). Several bioinformatics approaches have been used to identify microbial gene essentiality and putative new classes and functions for unique microbial genes in the metagenomic databases. HUMAnN is a program for metagenomic functional reconstruction to directly associate community functions with habitat and host phenotype. This program has been used to compare functional diversity and organismal ecology in the human microbiome (Abubucker et al., 2012). About 20% of all genes in a strain are essential and this has gained interest in drug discovery research (Christen et al., 2011). *In vitro* transposition and genetic transformation of the wild-type bacteria using a transposon library is a reliable experimental approach to uncover gene essentiality (van Opijnen et al., 2009). ESSENTIALS is another software for rapid analysis of high throughput transposon insertion sequencing data and discovery of essential genes (Zomer et al., 2012).

The majority of unique targets found in microbes’ genomes are genes responsible for the metabolism of carbohydrates, amino acids, xenobiotics, methanogenesis, and the biosynthesis of vitamins and isoprenoids. These genes are either non-homologous or orthologous to those encompassed in human genome. Vitamin biosynthetic pathways constitute a major source of potential drug targets. Most bacteria synthesize thiamine *de novo*, whereas humans depend on dietary uptake. Folic acid (vitamin B9) is an indispensable cofactor, which plays a key role in the methylation cycle and in DNA biosynthesis. Enzymes of the folate biosynthesis pathway, for example, dihydrofolate reductase, have been an attractive pharmaceutical targets for inhibiting folate synthesis. Sulfanilamide and trimethoprim are examples of effective antimicrobials used in a broad range of infectious diseases. Niacin (vitamin B3) participates in the biosynthesis of nicotinamide adenine dinucleotide (NAD^+^), a coenzyme essential in electron transport reactions in cell metabolism processes. Bacterial NAD+ kinases have been explored as targets for inhibiting bacterial growth. Methionine is not synthesized *de novo* in humans, and is supplied by diet. In contrast, most bacteria need to synthesize methionine to survive. *S*-adenosylmethionine synthetase, a key enzyme in methionine biosynthesis, is one drug target whose great potential has been explored against various pathogens. New drugs, for example platensimycin and platencin, that inhibit the microbial fatty acid synthesis (FAS) pathway by targeting key FAS enzymes have been successfully developed (Parsons et al., 2014). A recent survey identified 127 orthologous groups conserved in both human and human commensal gut microflora that are not suitable targets for drug development. However among these, the 20 aminoacyl-tRNA synthetases (aaRSs), which encode essential enzymes for protein synthesis, can be used since bacterial and eukaryotic AaRS have different specificity for tRNAs (Ochsner et al., 2007; Mobegi et al., 2014). These are only few examples of attractive targets for drug development; however, metagenomic data will open new frontiers for discovery of essential genes.

### Targeting Antibiotic Resistance Genes

The structure of the microbial community is maintained by specific microbial communication, cell signaling through cell-to-cell contact, metabolic interactions, and quorum sensing (Wright, 2010). Species within a bacterial community are either susceptible or resistant to epithelial innate AMPs and/or chemical antibiotics (Seo et al., 2010; Wozniak and Waldor, 2010; Sommer and Dantas, 2011). Bacterial genomes acquired resistance and metabolic genes from mobile genetic elements (MGE), including conjugative transposons, also called integrative conjugative elements (ICE), which are horizontally transferred by bacteriophages and plasmids (Wozniak and Waldor, 2010). Antibiotic resistance genes encoded in microbial genomes include multidrug eﬄux transporters, tetracycline resistance genes, vancomycin resistance genes, and beta-lactamases. In addition, a number of microbial genes and products, including bacteriocins, lysins, holins, restriction/modification endonuclease systems, and other virulence factors contribute to resistance to antibiotics (Dawid et al., 2007; Seo et al., 2010; Wozniak and Waldor, 2010; Smillie et al., 2011). Targeted (PCR-based) and functional metagenomic approaches have been used to track the presence of resistance genes or their families in different ecosystems (Mullany, 2014). A method to specifically trap plasmids containing antibiotic resistance genes called transposon-aided capture (TRACA) has been developed (Jones and Marchesi, 2007; Mullany, 2014). In this method, the plasmids are tagged with transposons that contain a selectable marker and a replication origin, which facilitate acquisition of plasmids from the human gut metagenomic DNA extracts, and subsequent maintenance and selection in an *E. coli* host.

Most of the antibiotics used to fight bacterial infections today are derived from soil microbes. Penicillin, the first true antibiotic, came from the soil fungus *Penicillium* (Kardos and Demain, 2011). To investigate the role of soil microbiota as a reservoir of genes encoding antibiotic resistance in the metagenomic data set, the ORFs found on contigs and on unassembled reads were compared with 3,000 known antibiotic resistance genes (Forsberg et al., 2014). It was concluded that most of the identified soil bacteria resistance genes were not typically close to known human pathogen resistance genes, suggesting little sharing between soil and gut bacterial species. A study on the microbiome of uncontacted Amerindians, members of a Yanomami isolated village living in the Amazon region has revealed the highest diversity of bacteria and genetic functions in fecal, oral, and skin bacterial microbiome ever reported compared with the US group (Clemente et al., 2015). Despite their isolation and no known exposure to commercial antibiotics, they carry functional antibiotic resistance genes with over >95% amino acid identity to those that confer resistance to semisynthetic and synthetic antibiotic monobactam and ceftazidime (Clemente et al., 2015). This finding provided important insights into how westernization impacts on the heritability of the microbiome among populations (Yatsunenko et al., 2012). There is evidence suggesting that exposure to microbes from animal gut microbiomes and within our indoor spaces (house, office, schools, cars, etc.) may become new sources for antibiotics and antibiotic resistance genes to human populations (Wright, 2010; Sommer and Dantas, 2011; Forslund et al., 2013). These discoveries emphasize the importance of continued functional investigations on antibiotic resistance reservoirs in metagenomic data from isolated ancestral and modern populations with a given disease.

### Targeting Quorum Sensing Systems

The term “Quorum Sensing” (QS) indicates systems used by bacteria to communicate with each other in order to synchronize their gene expression activities and behave in unison as a group (Miller and Bassler, 2001; Waters and Bassler, 2005; Hense and Schuster, 2015). This mechanism controls the synthesis of secreted products, disease-causing virulence factors, and many metabolites, including bacterial antibiotics that target competing bacteria, and substances that suppress the immune system (Miller and Bassler, 2001; Waters and Bassler, 2005). Thus, an alternative to killing or inhibiting growth of pathogenic bacteria is targeting these key regulatory systems (Finch et al., 1998; Defoirdt et al., 2010). Metagenomic studies have identified the genetic and phenotypic diversity of quorum-sensing systems that co-evolved with pathogenic species (Joelsson et al., 2006; Kimura, 2014). QS system was first described in marine bacteria *Vibrio harveyi* and *V. fischeri*, which use LuxI and LuxR proteins to control the expression of the luciferase enzyme for emitting luminesce upon reaching a critical mass or “quorum” (Nealson and Hastings, 1979). These bacteria secrete in the extracellular environment a small molecule, an acylated homoserine lactone (AHL), called autoinducer 1 (AI-1), to communicate with members of the same species (intraspecific communication; Miller and Bassler, 2001; Waters and Bassler, 2005; Ng and Bassler, 2009). After its discovery in marine bacteria, QS systems have been identified in more than 70 different bacterial species, including *Streptococcus pneumoniae*, *Bacillus subtilis*, and *Staphylococcus aureus* (Miller and Bassler, 2001; Waters and Bassler, 2005; Ng and Bassler, 2009). The QS systems control not only bioluminescence, but also other cooperative processes such as sporulation, conjugation, nutrient acquisition, biofilm formation, bio-corrosion, and antibiotics and toxins (Waters and Bassler, 2005; Kimura, 2014; Hense and Schuster, 2015). Remarkably, bacteria not only can communicate with members of the same species, but they are also able to sense the presence of different species in a community (interspecific communication). This interspecific communication is performed using a second type of autoinducer (AI-2). Thus, while each bacterial species has its own AI-1 to talk intraspecifically, AI-2 is common to all Gram-negative and Gram-positive bacteria. In fact AI-2 is not a single molecule but rather it refers to a group of molecules belonging to the family of interconverting furanones derived from 4,5-dihydroxy-2,3-pentanedione (DPD), whose biosynthesis is under the control of the enzyme LuxS (Xavier and Bassler, 2003). Development of novel compounds able to disrupt QS mechanisms has been carried out in recent years. For example QS quenching enzymes like lactonases and acylases are able to degrade acylated homoserine lactone (Dong and Zhang, 2005). A series of compounds, named halogenated furanones produced by many microbial species, mostly belonging to the Proteobacteria, can interfere with AHL and AI-2 QS pathways in Gram-negative and Gram-positive bacteria (Manefield et al., 2002; Rasko et al., 2008; Kayumov et al., 2014). Identification of the chemical signals, receptors, target genes, and mechanisms of signal transduction involved in quorum sensing are essential to our understanding how bacterial cell-cell communication may be used in preventing colonization by pathogenic bacteria. More data from metagenomic and metabolomics studies will help to decode the bacterial cross-talk and microbiome-immune system interplay, and particularly, distinctive regulatory mechanisms.

### Targeting Dysbiosis

#### Fecal Transplantation

Antibiotics have been used to treat infectious diseases over the past century. However, it is clear that antibiotic treatment can render individuals more susceptible to infections (Dethlefsen et al., 2008; Forslund et al., 2013). High doses and frequent use of antibiotics can disrupt and destabilize the normal bowel microbiota, predisposing patients to develop *Clostridium difficile* infections. Up to 35% of these patients develop a chronic recurrent pattern of disease. Fecal bacteriotherapy is the transplantation of liquid suspension of stool from a donor (usually a family member) and has been used successfully in severe cases of recurrent *C. difficile* relapse (Gough et al., 2011; Rupnik, 2015). However, many problems exist with this therapy since it can increase the risks of transmitting other pathogens (Brandt and Reddy, 2011).

Fecal transplantation studies in mice showed that transferring the microbiota from lean and fat mice to germ-free mice induces greater weight gain in those receiving the microbiota from fat donors (Ley et al., 2006). The discovery of the link between lean-associated microbiome has opened new possibility of using transplanted microbiota to treat metabolic disorders in humans.

#### Probiotics and Prebiotics

Probiotics are defined as live microorganisms that ultimately improve the balance of the intestinal flora, thus fostering healthy gut functions through a healthy gut microbiome (reviewed in Gareau et al., 2010; Whelan and Quigley, 2013). There are several *in vitro* assays to validate the actual *in vivo* efficacy of probiotic microorganisms, which include specific biological criteria, such as resistance to low gastric pH and capacity to reach the intestines alive to exert beneficial effects on the human body (Papadimitriou et al., 2015). Probiotic microorganisms are mainly lactic acid-producing bacteria of *Lactobacillus* and *Bifidobacterium* genera. Other microorganisms, such as the yeast *Saccharomyces boulardii* and the bacteria *E. coli* Nissle 1917, *Streptococcus thermophilus*, *F. parausnitzii* and *Bacillus polyfermenticus* have also been investigated. The beneficial therapeutic effects and mechanisms of action of Lactobacilli and bifidobacteria in patients with gastrointestinal diseases have long been demonstrated (Ng et al., 2009). These probiotics can prevent or ameliorate clinical symptoms of irritable bowel syndrome, inflammatory and necrotizing enterocolitis and acute diarrhea (Ng et al., 2009; Gareau et al., 2010; Whelan and Quigley, 2013). It was found that they could regulate the balance of intestinal microbiota by physically blocking the adhesion of pathogenic species onto epithelial cells. This is directly mediated by means of increases in the production of a mucosal barrier by goblet epithelial cells (Etzold et al., 2014). In addition, they can regulate epithelial permeability by enhancing the formation of tight-junctions between cells (Ng et al., 2009). Their immune-modulatory effects are associated with a decrease in the production of pro-inflammatory cytokines, as well as the microbial peptides bacteriocins (Ng et al., 2009; Whelan and Quigley, 2013).

The use of probiotics is not limited to gastrointestinal disorders. Studies evaluating their application in dermatology, urology and dentistry have been increasing (Vuotto et al., 2014). *Bifidobacterium bifidum* has been used in the prevention and treatment of infantile eczema. Intra-vaginal administration of *Lactobacillus rhamnosus GR-1 and L. fermentum* RC-14 were shown to have a positive effect on the prevention of recurrent BV and candidiasis (Anukam et al., 2006; Vuotto et al., 2014). Consumption of probiotics can be effective in the prevention of dental caries and periodontal diseases (Pandey et al., 2015). The continuous consumption of Yakult’s *L. casei* strain Shirota (LcS), one of the most popular probiotics, in adequate amounts, may reduce the risk of cancers by modulating immune function (Ishikawa et al., 2005). Finally, the treatment of obese mice with *Bifidobacterium infantis* was shown to reduce the production of pro-inflammatory cytokines and white adipose tissue weight (Cani et al., 2007). The effect of the endogenous host microbiota on obesity and beneficial role of probiotics including *L. rhamnosus* and *gasseri* and *Bifidobacterium lactis* in the treatment of adiposity and obesity has been reviewed elsewhere (Mekkes et al., 2014). This is a new area under intense investigation.

Prebiotics are functional food ingredients that can change the composition and/or the activity of the colonic flora (Roberfroid, 2000; Roberfroid, 2007; Brownawell et al., 2012). The dietary supplementation with prebiotics can promote the growth of beneficial bacteria such as lactobacilli and bifidobacteria strains (Roberfroid, 2000, 2007). Poorly digestible carbohydrates (fibers), such as resistant starch, non-starch polysaccharides (e.g., celluloses, hemicelluloses, pectins, and gums), oligosaccharides and polyphenols are resistant to gastric acidity, gastrointestinal absorption, and non-digestible by hydrolysis by mammalian enzymes. Colonic bacteria through carbohydrate hydrolyzing enzymes and fermentation produce hydrogen, methane, carbon dioxide, and SCFA, which can affect host energy levels and gut hormone regulation (Slavin, 2013). The most commonly used prebiotics are fructo-oligosaccharides (FOS) and trans-galacto-oligosaccharides (TOS), for example inulin (Roberfroid, 2000). However, not all dietary carbohydrates are prebiotics (Roberfroid, 2007). Mixtures of probiotic and prebiotic ingredients have been used to selectively stimulate growth or activity of health-promoting bacteria. In conclusion, it appears that the therapeutic use of pro- and prebiotics will find more applications in the near future when large-scale clinical trials and metagenomic surveys will determine which microbes are active, which are damaged, and which may respond to a given prebiotic, probiotic or synbiotic (synergic association of probiotic and prebiotic) at the genomic level (Nagata et al., 2011).

### Phage Therapy and CRISPRs

Phage therapy consists of using bacterial viruses bacteriophages, (also known as phages) as antimicrobial agents (Sulakvelidze et al., 2001; Abedon, 2014). Bacteriophages attach to specific receptors present in the host membrane and then inject their genetic material into the bacterium. Viral proteins are then synthetized using the host’s translational machinery. Phage infection can result in lysis, lysogeny or resistance. Lytic bacteriophages induce host cell death and breakdown in order to spread the infection whereas lysogenic (or temperate) phages insert their genome into the host DNA. Resistance may be acquired during replicative cycles by gene transposition or recombination.

Phage therapy can potentially have beneficial impact on human microbiomes and host health (Koskella and Meaden, 2013). The host specificity greatly limits the types of bacteria that will enter into contact with a particular phage, therefore avoiding the elimination of non-pathogenic species (Koskella and Meaden, 2013). However in order to choose a specific phage to use as a therapeutic agent, it is necessary to know the pathogen causing a given disease. When this is not the case, the use of a cocktail of different species of phages would broaden the range of action but could also have a possible negative effect on the microbial communities (Chan et al., 2013). The synergistic use of phages and low dose of antibiotics, a strategy named Phage-Antibiotic Synergy (PAS), could be useful in certain clinical situations (Comeau et al., 2007).

Bacteria have evolved various mechanisms of defense against phage infections, which act at different levels. In fact they can prevent phage attachment by mutation/loss of membrane receptors or block phage DNA entry with the aid of specific membrane proteins. Furthermore, Bacteria and Archaea developed an intrinsic innate immunity mechanism, which allows them to remember phage infection by capturing short DNA sequences from phage genetic material. These viral sequences are integrated as spacer sequences into their own chromosome, specifically into an array of repeated sequences called Clustered Regularly Interspaced Short Palindromic Repeats or CRISPR, with the help of the proteins encoded by Cas (CRISPR-associated) family of genes (Garneau et al., 2010; van der Oost et al., 2014).

CRISPR loci consist of short (~24–48 nucleotides) repeats separated by similarly sized, unique spacers found in genomes of Archaea (~90%), and Bacteria (~40%) (Garneau et al., 2010; van der Oost et al., 2014). Cas genes encode a large and heterogeneous family of proteins with functional domains typical of nucleases, helicases, polymerases, and polynucleotide-binding proteins. Upon invasion, the host organism samples and integrates in its genome short fragments of the foreign DNA, called protospacers, thus creating immunity against that particular infective agent. The protospacer is flanked by the repeated regions, and transcribed with them into a CRISPR-RNA (crRNA), which guides specific nucleases to a target DNA containing regions complementary to the protospacer. Upon recognition, nucleases cleave invasive DNA preventing it to replicate and blocking infection. Three different types and eleven subtypes of CRIPSR/Cas system can be classified based on their Cas protein repertoire and mechanisms of action (Plagens et al., 2015). A detailed list of type I, II, and III CRISPR-Cas systems is also available at the CRISPRdb website^7^. Type I systems are characterized by the molecular machinery named a Cascade complex (CRISPR-associated complex for antiviral defense) which displays nickase and exonuclease activities. Type III systems are characterized by the presence of Cas10 (the signature protein) and associated proteins. The systems are subclassifed as type III-A (CSM) and type III-B (CMR), depending on their specificity for DNA or RNA targets. In addition, types I and III share a variable number of repeat associated mysterious protein (RAMP) subunits (Rouillon et al., 2013; Plagens et al., 2015).

Type II is the simplest CRISPR-Cas system that is characterized by the presence of dsDNA endonuclease Cas9 and the transactivating CRISPR-RNA (tracrRNA). The tracrRNA anneals with the invariable regions of mature crRNA creating RNA heterodimers which, in turn, forms a nucleoprotein complex with Cas9, guiding it to the target DNA. Cas9 recognizes and binds to a specific 5′-NGG-3′ motif, called protospacer adjacent motif (PAM). Then the complex searches for a sequence complementary to the spacer portion of crRNA. Cas9 contains two nuclease domains, namely RuvC and HNH, and produces a double strand break in the target. Subsequently, cleaved DNA becomes a substrate of the bacterial DNA repair mechanisms, either non-homologous end joining (NHEJ) or homologous recombination (HR). NHEJ is an imperfect repair system and may cause insertion or deletion (indels) of base pairs, as well as single nucleotide polymorphisms (SNPs). However, high-fidelity HR repair may occur if a sequence complementary to the cleaved fragment is provided. The relative simplicity of the mechanism of action and the peculiarities of Cas9 make the CRISPR/Cas9 system an ideal tool for a vast assortment of procedures, particularly for genomic editing (reviewed in Ma et al., 2014; Selle and Barrangou, 2015; Xiao-Jie et al., 2015). A considerable amount of work in this field has been already done in different organisms, especially eukaryotes, using engineered versions of CRISPR/Cas9. On the other hand, despite its enormous potential, manipulation of bacterial genomes by CRISPR/Cas9 has so far been scarcely executed (Selle and Barrangou, 2015). CRISPR/Cas9 can be used to selectively deplete a given bacterial community of a particular harmful strain or species (Vercoe et al., 2013; Gomaa et al., 2014; Yosef et al., 2015). It has been shown that there is an inverse correlation between the presence of CRISPR loci and acquired antibiotic resistance in *Enterococcus fecalis* (Palmer and Gilmore, 2010), indicating that the use of antibiotics may increase the ability of bacteria to acquire drug resistance-encoding plasmids. CRISPR/Cas9 system can be used to introduce specific mutations into essential, antibiotic resistance, and virulence genes. It has been already shown that by providing *in trans* a DNA (linear or plasmid) homologous to the target sequence, it is possible to introduce very specific mutations to the desired target (Marraffini and Sontheimer, 2008; Jiang et al., 2013; Yosef et al., 2015). Also CRISPR/Cas9 has the potential to directly modulate the expression of particular genes. An engineered version of Cas9 lacking the nuclease activity but still retaining its binding capacity (dCas9) has already been created to repress bacterial transcription by binding to promoter regions or within a ORF, thus blocking transcriptional initiation and elongation, respectively. dCas9 can also be fused to regulatory domains in order to switch on/off the expression of specific genes (Bikard et al., 2013; Qi et al., 2013). In the near future the engineering of commensal bacteria with improved properties using a CRISPR/Cas system may constitute an effective vaccination tool in public health for prevention of diseases. However, despite great advances, still much work needs to be done in order to improve target specificity and delivering efficiency.

**FIGURE 2:**
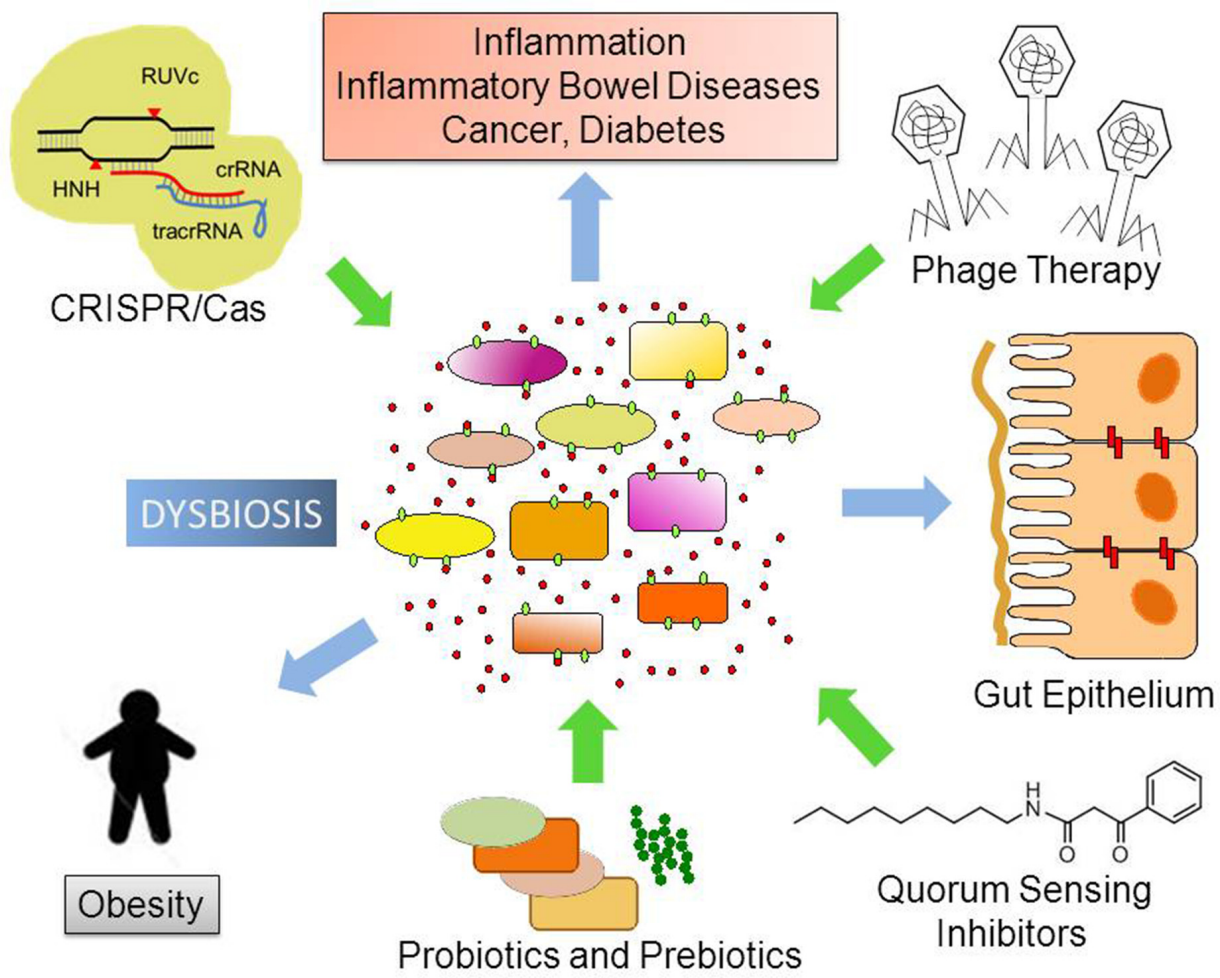
**Possible future therapeutic approaches to control dysbiosis.** Disturbances in the ecological community of commensal, symbiotic, and pathogenic microorganisms may favor dysbiosis. This leads to increased bacterial translocation and/or release of microorganism-associated molecular patterns (MAMPs), which activate Toll-like receptors (TLRs) in several cell types. Local and body-wide immune system activities promote inflammation, which ultimately leads to chronic diseases. Novel therapeutic approaches like phage therapy, disruption of Quorum Sensing and the use of the biotechnological tool CRISPR/Cas9 to edit microbial genomes have the potential to target specific bacterial taxa thus helping to re-establish homeostasis and microbiome balance.

A more complete perspective on how phage therapy and CRISPR/Cas9 systems can be employed to combat pathogenic species within our bodies, especially antibiotic-resistant bacterial pathogens needs the expansion of *in vitro*, *ex vivo*, and *in silico* approaches (Fritz et al., 2013). Several publicly available methods for hit-specific retrieval of protospacers in the reference microbiomes have already been developed (Bi et al., 2012). Over 123,003 protospacers have been predicted based on 690 phage genomes (Zhang et al., 2013). The functional exploration of pathogen-specific bacteriophages and gene therapy depends on development of relevant animal models including transgenic and bacteria-free animals (Fritz et al., 2013). Finally, we will need to confirm the results in the proof-of-concept in well-designed clinical trials. In **Figure 2**, we graphically summarize some of the pharmacological approaches discussed in this article.

### Ecopharmacology

To assess the interaction of the human body with pharmaceuticals, we need to understand the complex relationship between ecology, physiology, and pharmacology (Rahman et al., 2007; Flintoft, 2012; Haiser and Turnbaugh, 2012). From pharmacogenomic studies it is clear that sequence variations in drug target proteins, drug-metabolizing enzymes, and drug transporters can alter drug efficacy, produce side effects, causing variable drug responses in individual patients (Wilson and Nicholson, 2009). Microorganisms participate in a very wide range of biotransformations, including hydrolysis, and processing of glutathione conjugates of xenobiotics excreted in the bile (Johnson et al., 2012). Hence, the determination of the genetic variability of human microbiomes has potential to predict the efficacy, bioavailability and individual response variability in drug therapy.

**FIGURE 3:**
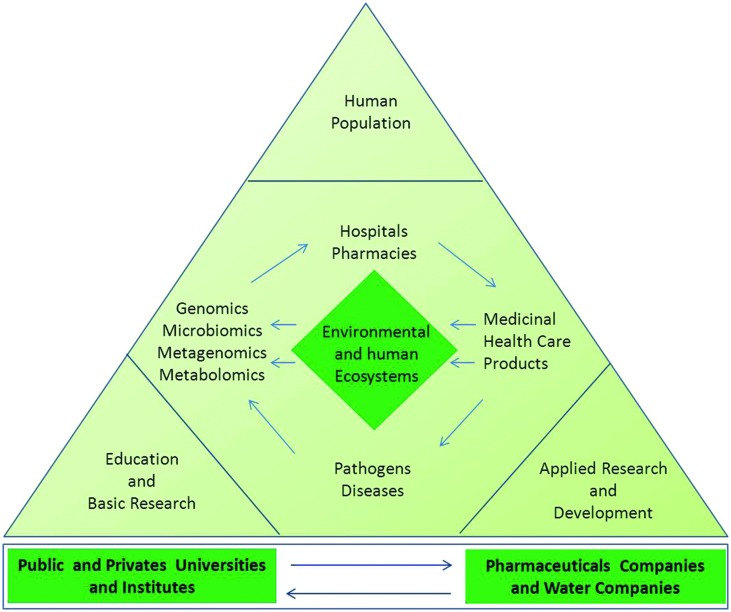
**Actions and molecular approaches aiming to protect the environmental and human microbial ecosystems.** The measurements of ecological, phylometagenomic, and microbial metabolic variations in the microbiomes require a specialized and complex set of knowledge. Collaboration between universities, research entities, non-governmental organizations (NGO), and the pharmaceutical industry professionals are key for evaluating both biological and pharmaceutical impacts in the ecosystems and elucidating the mechanism-of-action of new compounds in the host and its microbiomes. The utility of metagenomic functional reconstruction for direct association of community functions with habitat and host phenotype will be critical for proper study designs and production of greener pharmaceutical products for future personalized medicine.

Finally, further studies are needed to elucidate whether the vast number of functional microbiota gene-products exerts unknown off-target effects and how they can negatively or positively affect drug responses. These are the major research challenges for exploring the potential of metagenomics to better understand microbial ecology and to translate the molecular and genomic data into pharmacomicrobiomics (Saad et al., 2012). According to this new ecological paradigm, competency in knowledge, skills, and attitudes as well as integrated environmental conscience and social responsibility are essential for professionals who will in the future create and develop a new generation of green and sustainable pharmaceutical products, as shown in **Figure 3**.

## Conclusion and Perspectives

Recent advances in microbiome sequencing projects revealed the high complexity of microbial communities in various human body sites. They have confirmed the critical roles of the human-microbiota ecosystems in health-promoting or disease-causing processes. These studies have highlighted the unexpected and wide-ranging consequences of eliminating certain bacteria living in our body.

While the natural variation of the human microbiota has yet to be fully determined, the annotation and analyses of a large number of human microbiomes have shown that the presence or absence of specific microbial species categorizes human individuals based on enterotypes. It is likely that cultivated and uncultivated microbes will contribute to discovering new fundamental biomarkers for specific human disorders and that they may become better discriminatory tools than human-based ones.

Changes in the stability and dynamic of numerous microbial communities have been associated with several diseases, including type II diabetes, obesity, fatty liver disease, irritable bowel syndrome, and IBDs and even certain cancers. However, further studies need to be done in order to confirm whether low bacterial diversity increases the chances to develop such diseases and metabolic perturbations.

The use of antibiotics compromises genome defense and increases the ability to acquire antibiotic resistance. Prebiotics, probiotics, synbiotics, phage therapy, quorum sensing systems, and CRISPR/Cas systems have been proposed as tools to control and modulate microbial communities. Engineering of pathogen-specific bacteriophages and production of pharmaceuticals based on our own body’s microbiome will be possible and fully explored in the near future. The use of novel pharmaceuticals and nutraceuticals to modulate microbial colonization and development of a healthy gut microbial community in early childhood will support healthy adult human body functions and prevent the occurrence of several diseases.

## Author’s Contribution

The authors conducted the literature review process, grading, and categorizing criteria, and quality of selected articles. The authors read and approved the final manuscript.

## Conflict of Interest Statement

The authors declare that the research was conducted in the absence of any commercial or financial relationships that could be construed as a potential conflict of interest.

## Footnotes

1 http://www.human-microbiome.org/

2 http://www.arb-silva.de

3 http://www.metahit.eu/

4 http://www.hmpdacc.org/

5 http://www.genomesonline.org

6 http://vmc.vcu.edu/

7 http://crispr.u-psud.fr/
